# Treatment of Coral Wounds by Combining an Antiseptic Bilayer Film and an Injectable Antioxidant Biopolymer

**DOI:** 10.1038/s41598-020-57980-1

**Published:** 2020-01-22

**Authors:** Marco Contardi, Simone Montano, Giulia Liguori, José A. Heredia-Guerrero, Paolo Galli, Athanassia Athanassiou, Ilker S. Bayer

**Affiliations:** 1 Smart Materials, Istituto Italiano di Tecnologia, Genova, Italy; 2 Department of Earth and Environmental Sciences (DISAT), University of Milan – Bicocca, Milan, Italy; 3 MaRHE Center (Marine Research and High Education Center), Magoodhoo Island, Faafu Atoll, Republic of Maldives; 4 IHSM La Mayora, Departamento de Mejora Genética y Biotecnología, Consejo Superior de Investigaciones Científicas, Málaga, Spain

**Keywords:** Ecology, Materials science

## Abstract

Coral reefs are vital for the marine ecosystem and their potential disappearance can have unequivocal consequences on our environment. Aside from pollution-related threats (changes in water temperature, plastics, and acidity), corals can be injured by diseases, predators, humans and other invasive species. Diseases play an important role in this decline, but so far very few mitigation strategies have been proposed and developed to control this threat. In this work, we demonstrate that recently developed bi-layer human skin wound treatment patches containing antiseptics and natural antioxidants with controlled-release capacity can be adapted to treat scleractinian coral wounds effectively. A hydrophilic bilayer film based on polyvinylpyrrolidone (PVP) and hyaluronic acid was used to cover the open wounds while delivering the antiseptics for rapid action. Afterwards, the hydrophilic bi-layer covered wound was sealed with an antioxidant and hydrophobic ε-caprolactone-p-coumaric acid copolymer by melt injection at low temperatures. Treated coral injuries were monitored both in aquaria system and in natural environment in Maldives for over 4 months to reduce the number of entry points for organisms that could lead to diseases. The corals well-tolerated both biomaterials as well as the antiseptics incorporated in these materials. The treatments displayed self-adhering properties, tuneable dissolution time, and biocompatibility and stimulated regeneration properties within the coral wound. As such, this work demonstrates that certain human skin wound treatment materials can be successfully adapted to the curing of coral wounds and delivery of specific drugs to slow down, reduce or even stop the spread of diseases in scleractinian corals as well as in all other benthic organisms affected by uncontrolled pathologies.

## Introduction

Coral reefs are declining worldwide with coral diseases emerging as one of the most distressing threats^1^. In the last 50 years, this ecosystem is subjected to constant and extensive degradation, and a reduction of 25% of the global functional coral reefs has been recorded^2^. Among the coral reefs dwellers, reef-building corals are one of the most fragile components affected by changes in the marine realm^3^. Environmental stressors, including rising of the seawater temperatures, nutrient input of runoff, and sedimentation exacerbate the declining health of corals. Diseases play an important role in this apocalyptic scenario with the increased sea temperature that expedites the frequent diffusion of infections caused by microorganisms^3,4^. Currently, the number of known and identified coral diseases varies from 24 to 40, but it might be even more due to limited analytical characterization methods, deprived knowledge of the putative pathogens and the consequent deficiency of epizootiological data^5,6^. Therefore, the lack of well-defined knowledge makes the control of coral diseases a real challenge, especially when they are caused by bacteria^6^. One of the most dramatic and recent examples of such diseases is the Stony Coral Tissue Loss Disease (SCTLD), which has been destroying an entire ecosystem on the Florida Reef Tract since 2014. So far, its effect is unprecedented, and all the efforts carried out by researches have not been successful in discovering the relevant agents and taking the appropriate countermeasures^7^. For instance, mitigation techniques have already been proposed to reduce or slow down the progression of many coral diseases, however, most of them have not yielded and effective solution on a global scale^8^ yet.

In particular, coral wounds can be considered as the starting point of some coral diseases. Since the wound practically exposes the coral skeleton, pathogens settle on the skeleton and transform it into a proliferation substrate, increasing the likelihood of infections^9^. The first method to treat and manage a coral disease was developed in the late 1980s to control an outbreak of black-band disease (BBD) in Looe Key National Marine Sanctuary^10^ (lower Florida Keys, USA). Later on, Hudson *et al*.^10^ developed an aspirator device to remove the filament mats produced by the BBD bacterium, and then they covered the wound with a clay-based sealant. However, as they reported, in some cases, this passive covering did not ensure efficient disinfection of the underlying area leading to post-treatment infections of the corals. In the meantime, several other treatments and strategies have been proposed to manage and mitigate the damage inflicted by BBD and similar syndromes^11,12^. One of the most common strategy suggested was the removal of the lesion, considered as a common form of medical intervention to cure some diseases affecting both vertebrates and invertebrates^11,12^. Other than this, researchers used biological control techniques such as probiotics and phage therapy to manage bacterial pathogens^13^, the mechanical removal of disease vectors, such as the corallivore *Coralliophila abbreviata*^14^, and the excision of healthy coral branch tips from diseased *Acropora cervicornis* colonies^15^. In addition, researchers also used epoxy adhesives to mechanically block the progression of a tissue-loss disease on *A. cervicornis*^15^. Aeby et al. reported the application of antibacterial chlorine powder embedded within epoxy to treat and mechanically block the progression of BBD in *Montipora*^12^, but considering the polymicrobial origin of several coral diseases, the employment of more specific type of drugs can be a better strategy to complete eradicate the infection.

A recent work of Sweet *et al*.^16^ showed as some antibiotics can be employed for the treatment of the White band disease (WBD), a polymicrobial infection of the corals. Here, the authors simply added the antibiotics at a suitable concentration in the water of the aquarium. Although the results were positive in mitigating the WBD diffusion, the same authors highlighted how this type of treatment could not be applied at a large-scale level. Indeed, to reach an effective concentration of antibiotic in the sea, we would risk compromising the marine ecosystem, limiting this approach to emergency cases in the aquarium environment.

Therefore, the ever-increasing spread of coral diseases and the lack of adequate healing strategies bring up the urgent necessity to design new treatments for the *in situ* delivery of drugs such as antiseptics, antibacterial agents or anti-protozoans, and, at the same time, avoiding the spread of potential pollutions in the sea.

One of the approaches could be the fabrication of biocompatible, biodegradable, and bioactive polymeric drug delivery systems suitable for the coral application. In particular, the modern strategies and treatments applied for human wounds can be a point of inspiration and constitute some potential guidelines also for the coral disease prevention^17–20^. In humans, the main role of the skin tissue is to protect the body from external pathogenic agents, acting as a shield^21^. After a skin injury, the loss of this barrier can cause rapid infections and bacterial colonization. The presence of bacteria in the wound bed is one of the reasons why the healing process is delayed^21^. Therefore, in order to avoid bacterial infection and promote the healing, an ideal wound dressing, firstly, should ensure a covering of the wound. At the same time, the wound dressing should be able to kill the bacteria that have already infected the wound^22^. Similarly, for coral injuries or wounds, the potential wound dressings must be made from materials that are not bio-persistent and toxic for marine environment in general. Intelligent wound dressings can be made from several polymers and active compounds that can be infused with medicinal compounds to accelerate and manage the healing process. Among them, the biocompatible synthetic polymer polyvinylpyrrolidone (PVP) has gained interest in the topical application not only because it can modulate the crystallinity of several drugs favourably but also for its capacity to form transparent films and ensure good self-adhesion to the moist skin^23–25^. In recent years, the natural polymer hyaluronic acid (HA) has also played an important role in the pharmaceutical field due to its anti-inflammatory, regenerative and anti-age properties. By the same token, polycaprolactone (PCL) is a biodegradable synthetic polyester^26^ that has been broadly applied in biomedical fields for the preparation of controlled drug delivery systems such as implants and scaffolds^27,28^. It has been recently combined or co-polymerized with effective natural antioxidants such as vanillic, ferulic, p-coumaric and syringic acids^29^.

In this work, we attempted to use polymeric smart wound dressings in tandem, inspired by human skin wound care and management to cure coral wounds. In particular, we used a dual-drug carrying PVP-HA-based bilayer construct, loaded with eosin-based antiseptic and the antibiotic Ciprofloxacin, as well as a ε-caprolactone-p-coumaric acid copolymer (PCL-PCA) developed for skin regeneration^25,30^.

**Figure 1:**
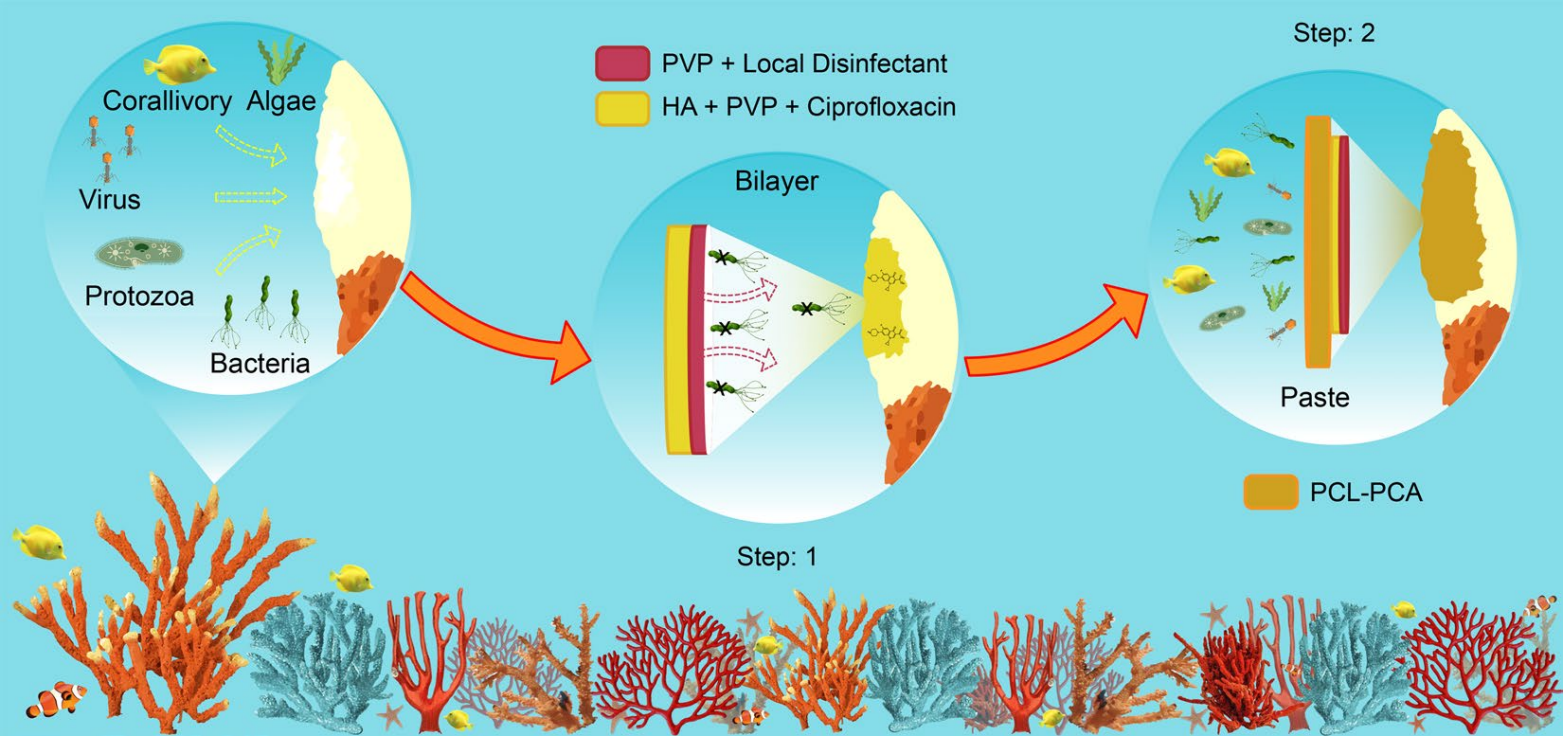
Schematic representation of a coral wound and its potential treatment. Illustration of a coral wound in the sea. In the first close-up, all the potential risks when a coral loses its protection are presented. In the other two close-ups, the two potential steps of the treatment suggested in this work are shown. The bilayer should act as a bioactive dressing for the delivery of drugs in order to disinfect and treat the wound (Step 1). Instead, the PCL-PCA paste should act as a shield against all the external agents and the fast dissolution of the bilayer dressing (Step 2).

Dual drug-carrying bilayer construct was applied directly onto the wound and acted as material for the *in situ* delivery of two antibacterial drugs^25^ (see Fig. 1). Afterwards, the PCL-PCA copolymer with antioxidant capacity was melted to seal and shield the wound. The two materials were applied and tested in combination (see Fig. 1) and separately as control samples, and monitored during two distinct phases: *ex-situ* and *in-situ*. The first part of the experiments consisted of 10-days of observation *ex-situ*, and we focused on understanding the ability of the two materials to adhere to the coral surface, to resist underwater dissolution and to evaluate potential toxic effects on the coral, if any. Afterwards, the survival and the health of the corals were followed for four months continuously *in-situ*.

## Results and Discussion

**Figure 2:**
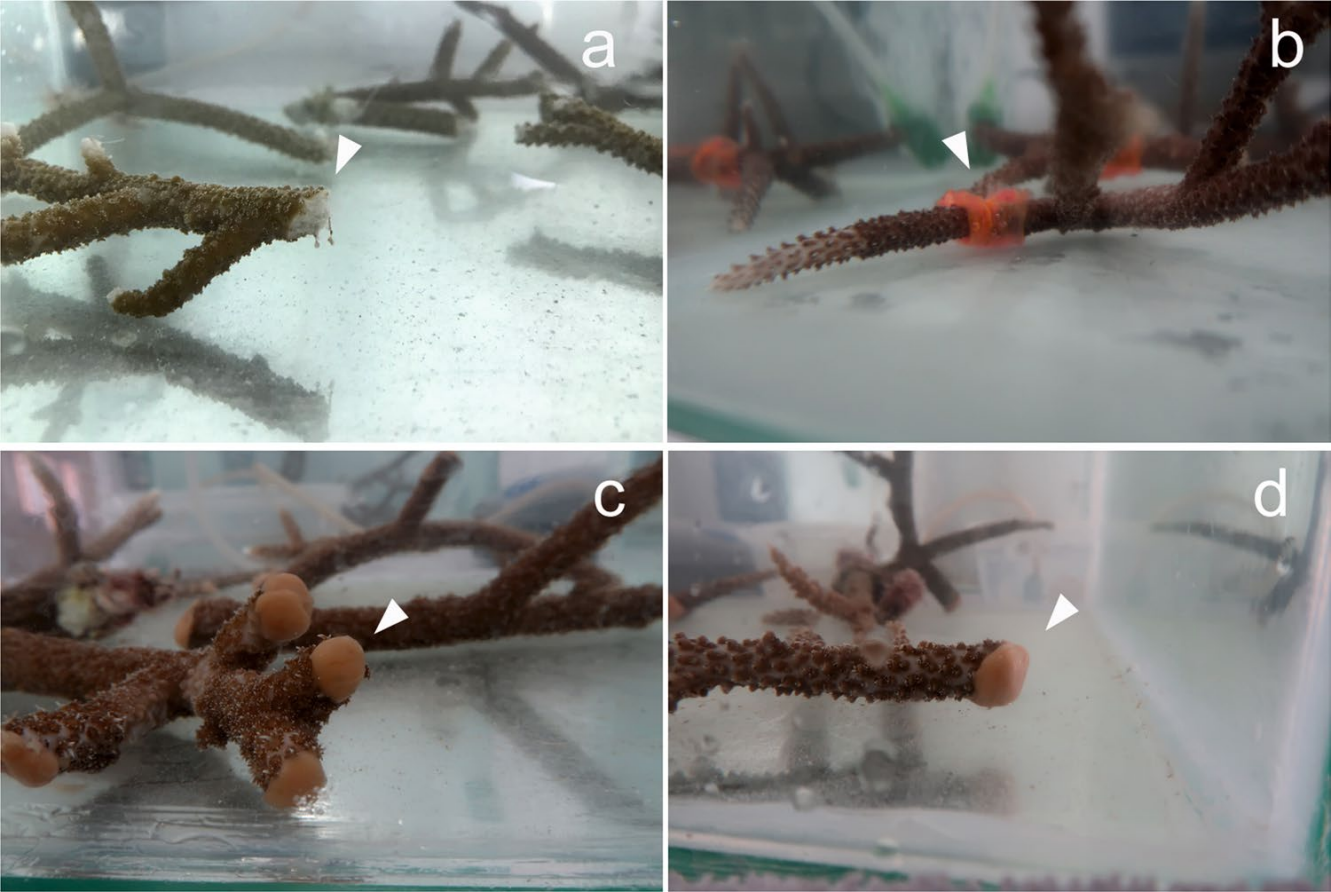
Coral wounds. Photographs in tank immediately after inflicted injuries of the control (**a**), bilayer-treated sample (**b**), PCL-PCA-treated sample (**c**) and bilayer + PCL-PCA-treated sample. (**d**) The arrows indicate the position of the injuries.

Coral wounds may increase susceptibility to some diseases^31^, hence, the materials developed and used in this study are expected to prevent disease outbreaks in the short term relevant to the early stages of the coral wound. During the monitoring period, the applied materials were well-tolerated by the coral system and exhibited proper and healthy restoration properties. In total, 52 coral wounds were created and they were monitored within 4 experimental groups: untreated, treated with only bilayer, treated with only PCL-PCA copolymer, and treated with both bilayer and PCL-PCA copolymer (Figs. 2 and S1).

**Figure 3:**
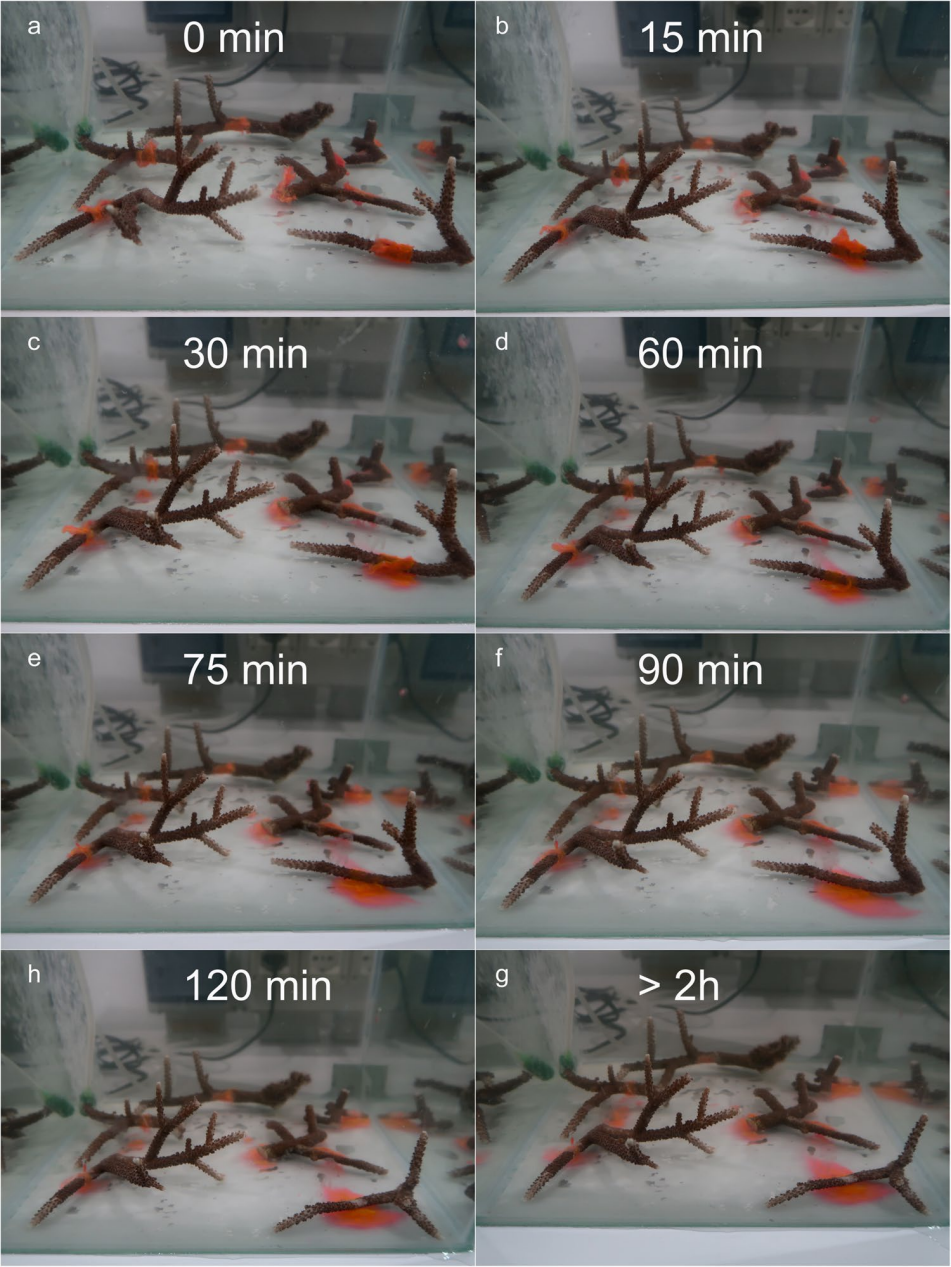
Application of bilayer materials on coral wounds. Photographs of bilayer applied on different coral wound monitored for the first 3 hours.

The first treatment tested consisted of the application of the dual drug-carrying PVP-HA-based bilayer film directly on the open coral wounds. In total, 8 lesions have been treated with this material, where each of them has been carefully covered by a strip of bilayer film wrapped over the wound (red-colored foils in Fig. 3a). As expected, due to the hydrophilic nature of its components PVP and HA, the bilayer dissolved and disintegrated in few hours after the total immersion in the water. The dissolution process started about 10–15 minutes after the immersion (Fig. 3b) in all coral-induced wounds where the material has been located. Eventually, the bilayer film disappeared, revealing the bare skeleton underneath after about 3 hours (Fig. 3b–g). The treatment has been monitored for 10 days to observe any further changes on the coral fragments before termination.

For the other 3 experimental groups, during this *ex-situ* phase, we took into account different parameters, reported in Table S1, to investigate the state of health of the corals. In particular, we evaluated the injury condition, the colony health, and the biopaste condition. For each condition, three variables were ranked with values from 0 to 4 (see Table S1).

**Figure 4:**
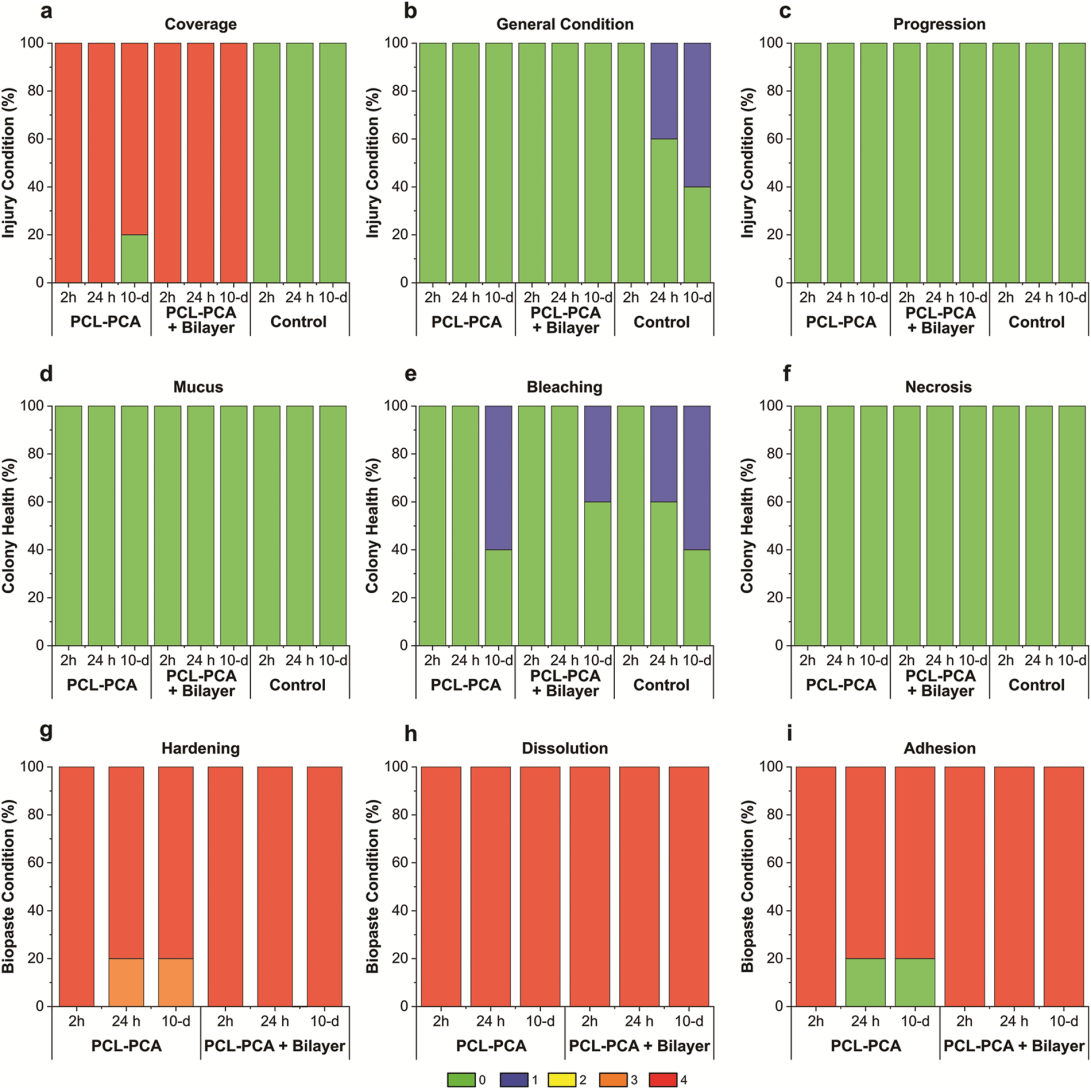
Monitoring parameters. Graphs of the observed parameters during the *ex-situ* phase for the PCL-PCA, PCL-PCA + Bilayer and for the Control expressed as “Injury Condition” (**a–c**), “Colony Health” (**d–f**), and “Biopaste Condition” (**g–i**).

In Fig. 4, we reported the main results of the monitoring parameters at different time points. For the injury condition, we reported results about the percentage of covered injuries, the general condition of the injuries, and the progression of the healing. As colony health, we evaluated the formation of mucus, the bleaching, and the necrosis. Instead, regarding the biopaste condition, we monitored the hardening, the adhesion, and the dissolution parameters.

**Figure 5:**
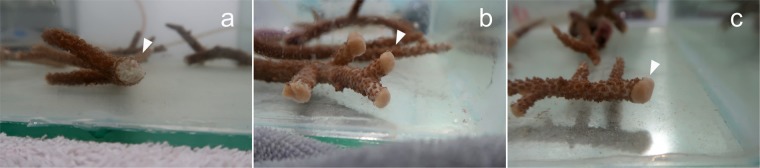
Coral wounds status after 10 days. Photographs in tank after 10 days from the injuries of the control (**a**), PCL-PCA sample (**b**) and bilayer + PCL-PCA sample. (**c**) The arrows indicate the position of the injuries.

The PCL-PCA copolymer treatment consisted of the application of a melted material directly on the 16 induced coral wounds. The PCL-PCA copolymer is a solid film at room temperature, thus to obtain a conformal coverage of the coral wound, it was heated to 60 °C and immediately applied in the molten state. After the immersion in water, the material solidified, leaving a cup-shaped self-adherent structure on all coral wounds (Fig. 5b). Immediately after the application and during the whole initial phase of monitoring period (10 days), the PCL-PCA copolymer remained adherent to the wounds and no signs of dissolution and detachment were noticed for all the coral wounds under analysis except for a single wound that lost few small pieces from the top portion of the cup without exposing the wound. Furthermore, even if no detectable anomalies have been observed regarding the mucus production and presence of necrosis tissue, a slight variation in color in three of 16 fragments was observed between day 8 and day 10. In particular, those fragments showed a slightly pale tissue diffuse spread throughout their surface (Fig. 4d–f).

Based on these observations, it appears that the application of the melt copolymer does not injure the coral. Note that the copolymer is not constantly kept at its melting point during its application on the coral. Hence, it already cools down during application. However, we are aware that the melted copolymer can prematurely close the wound or kill some bacteria. At this stage, it is difficult to quantify this. However, during the application, the wound does not experience a constant high temperature. In relation to this, some works^32,33^ demonstrated that a similar temperature maintained at a longer time can have a weak antibacterial activity. We cannot exclude that the molten biopolymer injection process can affect the microorganisms present in the wound. Hence, further in-depth studies will be planned and conducted to this effect.

For the third set of experiments, a dual treatment of bilayer and PCL-PCA was applied on 12 coral-induced wounds (Fig. 5c). This treatment combines the possibility of a short time *in situ* drug delivery ensured by the bilayer film and the sealing and shielding activity of the PCL-PCA cup. This tandem treatment demonstrated superior self-adhesion to the wound surface with respect to the PCL-PCA treatment. In support of this, none of the applied PCL-PCA structures came off the induced coral wounds and, moreover, no variations in the consistency or dissolution of the copolymer have been detected (Fig. 4g,h). In line with this, the condition of the lesion zones after 10 days of monitoring remained at an optimal state, with no changes in the coverage status and including the backside of the lesion zone. A small alteration observed was related to three fragments that appeared slightly paled between day 9 and day 10. No relevant signs of mucus production and the presence of tissue necrosis were noticed; see Figs. 4d,f and 5b,c.

On the untreated coral wounds, no significant changes occurred in the inflicted wounds during 10 days of monitoring (Fig. 5a). However, light pale conditions and patches over the wound surfaces formed almost on all the fragments monitored (Fig. 4e).

During these 10 days of indoor aquaria system monitoring, all fragments survived, and no clear differences were observed among the untreated control, the PCL-PCA and bilayer/PCL-PCA-treated samples in terms of healing or progression of the injuries, see Fig. 4. This may be attributed to the particular coral species and its resistance, the number of wounds produced, absence of infection, use of aquaria controlled systems and more likely to a combination of these scenarios. Moreover, to the best of our knowledge, several causes can trigger the host’s immune responses involved in wound healing such as the toll-like pathway, the melanin synthesis for tissue regeneration, the complementary system resulting in apoptosis of damaged cells, and cell activation to move amoebocytes to the wound^34^. Furthermore, tissue regeneration and its associated immune pathways require extensive cellular resources (e.g., carbon products and amoebocytes) to be translocated to the wound^35^. Therefore, to further explore the effectiveness of the treatments, in the second part of the study, all the coral fragments were attached on a rope and dipped 5–20 meters down the ocean waters (December 2018, Maldives) and monitored till April 2019 (see Figure S2). The *in-situ* monitoring revealed that all treated fragments survived and that most of the injuries showed complete tissue recovery after about 4 months.

**Figure 6:**
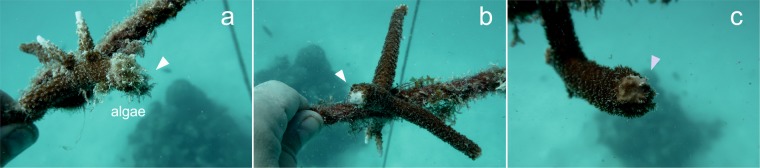
Coral wounds status after 2 months. Photographs underwater after 2 months from the injuries of the control (**a**), PCL-PCA-treated sample (**b**) and bilayer + PCL-PCA-treated sample. (**c**) The arrows indicate the position of the injuries.

By contrast, after 2 months of observations on untreated wounds (the control samples) demonstrated some lesions with algal overgrowth (see white arrow in Fig. 6a), suggesting a failure in the natural healing process compared to the treated samples where no algae growth and invasion were noticed (Fig. 6b,c).

**Figure 7:**
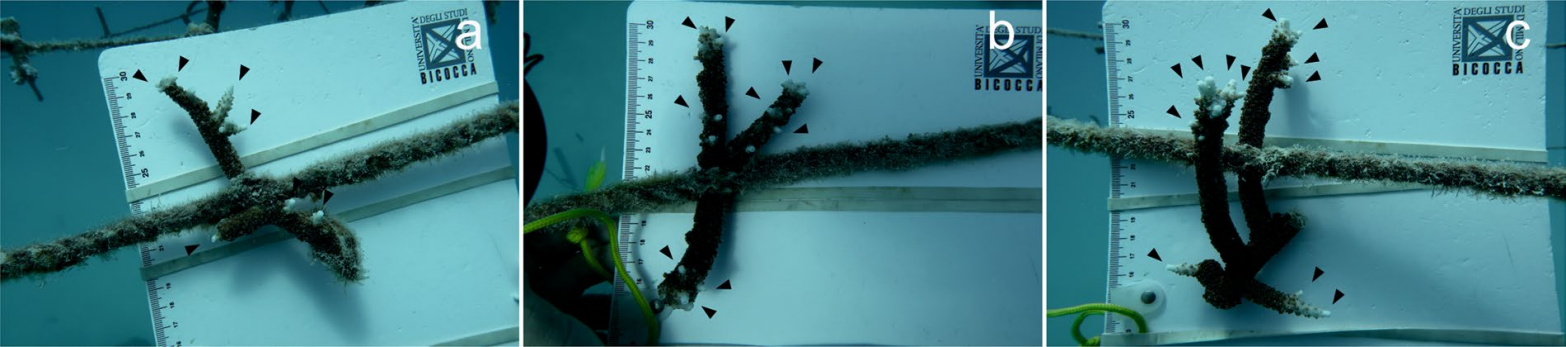
Coral wounds status after 4 months. Photographs underwater after 4 months from the injuries of the control (**a**), PCL-PCA sample (**b**) and bilayer + PCL-PCA sample (**c**). The arrows indicate the position of the new tips.

After 4 months underwater, we used the generation of new tips “branchiness” as measure of a positive healing effect on the coral fragments treated with PCL-PCA and bilayer/PCL-PCA materials and compared the results with untreated coral fragments. All fragments showed formation of new tips (including the control samples), confirming the suitability of Maldivian lagoons for the restoration of branching *Acropora* corals, see Fig. 7.

**Figure 8:**
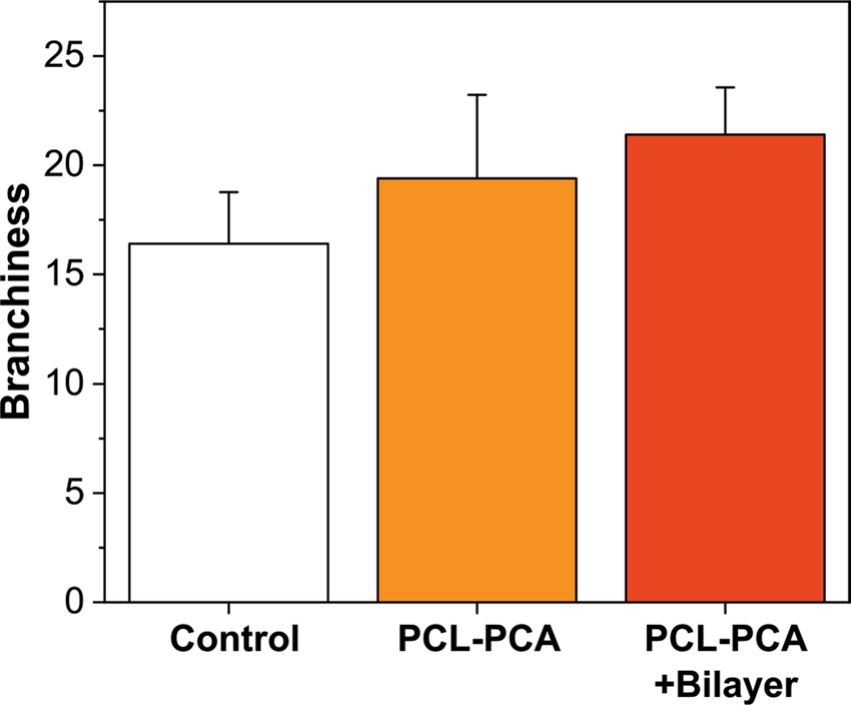
Branchiness. The number of new tips for fragment formed after 4 months from the wound injury.

An average of 16.4 new tips per fragment for the untreated corals was observed. Instead, in the case of corals treated with PCL-PCA and PCL-PCA/bilayer an average of 19.4 and 21.4 new tips per coral fragment were recorded, respectively (Fig. 8).

Although the branchiness per fragment was not statistically significant between the treatments (H- Kruskal Wallis p = 0.323), this trend was also observed in the total number of new tips formed. Indeed, the untreated coral produced in total 82 new tips, while the corals treated with PCL-PCA and PCL-PCA/bilayer, generated 97 and 107 new tips, respectively. Moreover, the highest number of new tips (n = 34) has been observed on a fragment treated with PCL-PCA/bilayer.

As such, although the tandem treatment appears to have a positive impact on the coral fragment restoration, the next steps will be focused on verifying the efficacy of the treatment in an infected coral system. However, the slight difference observed may be indicative of possible future development of similar tools for the large-scale treatment of coral diseases. In the real ocean habitat, the number of new tips may be influenced by many factors such as the number of starting branches, the total size of the fragment, the presence of predators, nonetheless, present observations indicate that the fragments treated with PCL-PCA/bilayer grow and heal faster than the untreated and PCL-PCA-treated samples. It might be possible that the fragments without treatments needed more energy to cover the injuries with the new tissue. In fact, corals use the same finite cellular resources for both tissue regeneration and reproduction^35^, creating a tradeoff between these metabolic processes. Immediately after the damage, polyps neighboring lesions experience a limited supply of cellular resources, reducing local reproductive output within the colony as resources are shunted toward wound healing^36^.

On the other hand, we cannot exclude that the competition between algae invasion or other organisms that colonize the open wound and healing may slow the recovery process in the control coral fragments compared with the other two treatments. In line with this, the use of injectable PCL-PCA copolymer as a seal for injuries not only closes the injury but may also hinder the settlement of other organisms, including the algae. Although more observations and data would be required, this may be attributed to the inherent antibacterial, antifungal and antioxidant properties of p-coumaric acid^37^. More habitat observations will be needed to confirm these preliminary but encouraging observations toward controlling coral reef damage. Similarly, finding the proper coral wound dressing treatments may not only reduce the corallivores activity but also may function to enhance coral growth. Indeed, protection from corallivores with various foraging strategies can increase coral growth^38^ and coral reproductive potential^39^. Moreover, beyond affecting corals through mechanical damage, corallivores provide entry points for opportunistic bacteria (e.g., coral diseases) and can aid in parasite transmission on the reef^40^. Further understanding of how the use of antiseptic bilayer and the injectable PCL-PCA copolymer might shape the effects of corallivores on coral health will also be important and will need to be elucidated in the next future.

It is widely acknowledged that the systems of large-scale coral reefs are declining^41^. Strategies for ecosystem-scale reefs conservation is now an indispensable component for the recovery of this ecosystem. Given the inability of these species^2^ to adapt to rapid environmental changes^42^, vigorous efforts have been initiated in various areas of the world to enhance the recovery of reefs or increase their resilience^43^. One example is the coral reef restoration practice that consists of the active recovery of an area that has been degraded, damaged, or destroyed by direct transplantation, gardening and seeding approach^44,45^. This requires open wound coverage to avoid settlement of pathogens or reducing the spread of infectious disease as well as the algal overgrowth. Thus, the use of new mitigation tools aimed to cure open wounds will be at the forefront of these efforts.

In conclusion, our preliminary tests reveal that treating scleractinian coral injuries/wounds by combining a rapidly dissolving antiseptic bilayer film with an injectable antioxidant copolymer can be a promising method to reduce the number of wound entry points for organisms that could lead to diseases. Scleractinian coral well-tolerated both biomaterials tested as well as the antiseptics incorporated in these materials. In addition, the treatments had very good self-adhering properties, tuneable dissolution time, and biocompatibility and promoted regeneration of coral tissues. PCL-PCA copolymer not only acts as a hydrophobic seal for the wound but also keeps the treated zone free from invasive proliferation. This work could be a first step toward applying active biomaterials developed for human wound treatment to the coral restoration field.

However, it is stressed that future work related to this tandem treatment should be conducted in more detail on already infected coral wounds in their real habitats in order to assess its efficiency. Nonetheless, these preliminary observations are encouraging in the sense that certain human skin wound treatment soft materials can be adapted to the sealing and subsequent curing cure of coral wounds while delivering specific drugs to the potential infection zones. This would pave the way to slow down, reduce or even stop the spread of diseases in scleractinian corals as well as in all other benthic organisms affected by uncontrolled pathologies.

## Methods

### Bilayer fabrication

The details of the preparation of bilayer wound dressing films were given in a recent study^25^. In summary, the first layer was easily obtained by solvent casting method starting from an acetic acid (AcOH)/water solution (1% v/v in AcOH) containing hyaluronic acid 2% (w/v), polyvinylpyrrolidone 2% (w/v), ciprofloxacin 0.014% (w/v), and glycerol (to a concentration of 10 wt% with respect to total polymer dry film weight). For the fabrication of the second layer, an aqueous solution based on PVP 20% (w/v), NEOMERCUROCROMO 10% (v/v) and glycerol (10 wt% dry basis) was spread on the first layer using two different methodologies: spin-coating and rod-coating. Here, we decided to use the rod-coating method because it allowed us to obtain larger size samples that can be easily adapted to different coral wound sizes. Therefore, samples of the first layer with a rectangular shape (15 × 30 cm) were prepared. Afterward, the second layer was fabricated using a Rod-coater system (model EZ coater EC-200; ChemInstruments) over the initial dry film.

### PCL-PCA copolymer synthesis

The synthesis of the ɛ-caprolactone-*p*-coumaric acid copolymers follows the protocol described by Contardi *et al*.^30^. Briefly, the monomer ɛ-caprolactone was melted and maintained at 150 °C and then *p*-coumaric and the catalyst 4-dodecylbenzene sulfonic acid (DBSA) were added. This mixture was stirred at 150 °C for 24 h. After that, the blend was cooled at room temperature to obtain a solid material. This step was followed by the dissolution of the solid material in 25 mL of chloroform and precipitation of the copolymers with an excess of cold methanol. Then, the polyesters were washed with water and methanol (50 mL per gram of film) three times each one and dried at room temperature for 24 h under vacuum. Finally, in order to form homogeneous films, the samples were melted at 75 °C for a few minutes and cast into Teflon Petri dishes at room temperature.

### Coral injuries preparation

The study was carried out between December 2018 and April 2019 in the marine laboratories facility at the Marine Research and High Education Center (MaRHE), placed in the Magoodhoo Island, Faafu Atoll, Republic of Maldives (see Figure S3). To test if the mitigation tool can be used in coral reef habitats, the staghorn coral *Acropora muricata* was chosen for this study since it represents one of the most abundant coral species in the Indo-Pacific reef^46^, including in the Maldives where their populations have severely suffered from intense mortality events due algal overgrowth^47^, and coral diseases^48–51^. Before sampling, the presence of *A. muricata* was recorded qualitatively by applying a roving dive technique with SCUBA, in which a 1 h dive served as the sampling unit, by starting at the maximum depth at each dive site (15–25 m) and moving from there to shallower water^52^. For documentary purposes, underwater photographs of *A. muricata* were taken using a Canon GX7 camera in a Fantasea underwater housing. Afterward that four colonies of the scleractinian corals *A. muricata*, of approximatively of the same size (30 × 30 × 20 cm), were sampled in the coral reefs surrounding Magoodhoo by SCUBA diving. Coral fragments (n = 5–6 per colony) were broken off with a side cutter, (approximately 8–12 cm in length), creating at the same time from 1 to 4 injuries of about one square centimeter (Fig. S1). Corals were further scratched to create additional open wounds on the coral surface if necessary. All coral colonies were identified at the species level, following the last revised taxonomic classifications for Acroporidae^53^.

### Experimental design

Before setting up the experiments, the coral colonies were acclimated for 48–72 hours in an aquaria system consisting of four 60-l seawater tanks, connected to a 330-l sump containing gravel-bed filter, protein skimmer and a 500 W Titanium Heater (Aqua Medic) connected to a temperature controller. In all the tanks, light was provided by 400 W metal halide lamps (Powerstar HQI-T, Osram), which were turned on at 9 a.m. for the day cycle and turned off at 9 p.m. (12:12 light:dark cycle).

The experimental design was divided into an *ex-situ* phase, conducted in standard controlled aquaria system (tank), and an *in-situ* phase, moving the samples in the ocean ecosystem (Maldives).

After the acclimation period, in the *ex-situ* phase, three different treatments (bilayer only, PCL-PCA only, Bilayer + PCL-PCA) plus control have been monitored. The treatments consist of (1) Bilayer, coral wounds have been covered by bilayer only. It was applied out of the water, gently covering as much possible the wounds until complete adhesion; (2) PCL-PCA, the PCL-PCA has a melting point of ≈50 °C, before application, it was kept at 60 °C in order to ensure a homogeneous liquidity and to delay rapid solidification during injection. Once the biomaterial was melted, the wounds were covered out of the water and then the coral fragments were put back in the tank (seawater at 27 °C); (3) Bilayer + PCL-PCA, it consisted in the application of both biomaterials on the induced coral wounds. In this case, the bilayer has been applied first, followed immediately by the PCL-PCA used to cover and seal the bilayer in the injuries. Finally, a tank with induced wounds on *Acropora muricata* without cure has been used as untreated. The condition of each single coral fragments, for each treatment, of both lesion and materials have been monitored every 15 minutes in the first two hours, then each hour for the next 10 hours, and then every 12 hours for the following 10 days.

After this period, the *in-situ* phase followed. Here, in order to see the efficacy of the treatments, the coral fragments were deployed in a rope on coral nurseries hanging at 5–20 meters’ depth (Fig. S2). In this phase, we collected data about the presence of new tips, herein called as “branchiness”, in order to assess the additional health condition of the fragments.

The branchiness was statistically compared between the treatments using the Kruskal-Wallis test because the data did not meet the normality assumption. Statistical analyses were performed using SPSS computer software (IBM, NY, USA). All data are presented as arithmetic means ± standard error (SE) unless stated otherwise.

### Statement of source of figures

The authors want to clarify the authorship and the source of the Figs. 1–3, 5–7, S1–S3. In particular, the Fig. 1 was realized by Dr. Marco Contardi by means of the software Illustrator. Instead the Figs. 2, 3, 5–7, S1, S2, S3 were assembled by Dr. Simone Montano using the software CorelDraw. In particulare the photographes were taken in Dr. Simone Montano’s laboratory and in the Maldives sea by means of a camera Canon G7X. Therefore, the authorship of the figures belongs to Dr. Marco Contardi and Dr. Simone Montano.

## Supplementary information

Supporting Information.

Supporting Information.
