# Supplementary material for: Treatment of Coral Wounds by Combining an Antiseptic Bilayer Film and an Injectable Antioxidant Biopolymer
Source: Sci Rep. 2020 Jan 22;10:988. doi: 10.1038/s41598-020-57980-1 (PMC6976594; doi:10.1038/s41598-020-57980-1)
Supplement: Supplementary file 1 — Supporting Information. [file 41598_2020_57980_MOESM1_ESM.pdf]

Supporting Information

Treatment of Coral Wounds by Combining an Antiseptic Bilayer Film and an Injectable Antioxidant Biopolymer

Marco Contardi[a,*], Simone Montano[b-c], Giulia Liguori[c], José A. Heredia-Guerrero[a], Paolo Galli[b-c], Athanassia Athanassiou[a], Ilker S. Bayer[a,*]

[a]*Smart Materials, Istituto Italiano di Tecnologia, Genova, Italy,*

[b]*Department of Earth and Environmental Sciences (DISAT), University of Milan – Bicocca, Milan, Italy.*

[c]*MaRHE Center (Marine Research and High Education Center), Magoodhoo Island, Faafu Atoll, Republic of Maldives*

* Corresponding authors: marco.contardi@iit.it (M.C.), ilker.bayer@iit.it (I.B.)

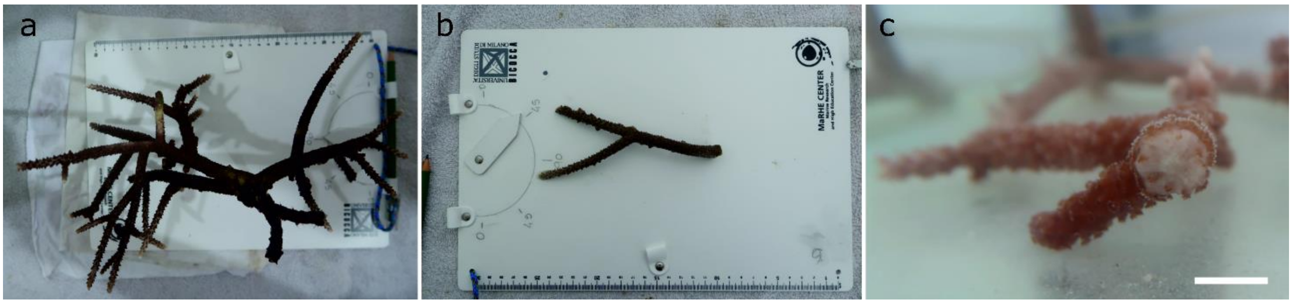

Figure S1. Coral characterization and induced wound. Photographs of coral colonies (a; b) and an induced coral wound (c).

Injury condition	Coverage	General condition	Progression
0	0	healthy	stable
1	25	not healthy (edges)	slighty increase
2	50	not healthy	huge increase
3	75	aggravation	tissue regrowth
4	100	treatment not efficent	total tissue regrowth

Colony Health	Muco	Bleaching	Necrosis
0	none	none	none
1	slighty	slighty pale (partial)	slighty close tips
2	diffused	pale (all colony)	diffused (other parts)
3	heavily diffused	white	total (all colony)
4	death	death	death

Bio-paste condition	Hardening	Dissolution	Adhesion
0	not applicable	not applicable	none
1	soft	istant dissolution	doubt application
2	semi rigid	rapid dissolution (24H)	not perfect
3	rigid	slow dissolution (10 gg)	adhesion lesion/no tissue
4	hard (plastic like)	no dissolution	adhesion lesion and tissue

Table S1. Monitoring parameters. The table reports all the parameters take into account during the first 10 days of observations.

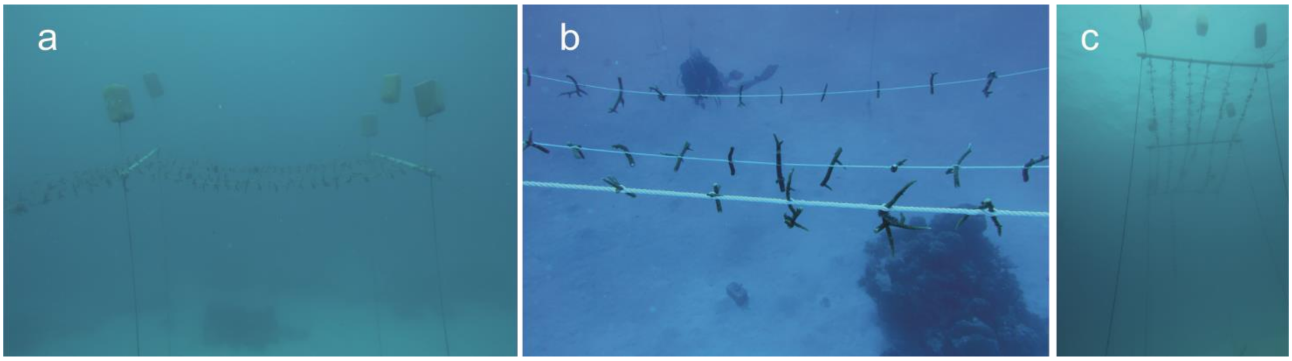

Figure S2. Photographs of the experimental site. The figure shows the underwater site where the fragments were deployed: a) coral rope nursery; b) rope with treated fragments; c) overview of the nursery and fragments from the bottom**.**

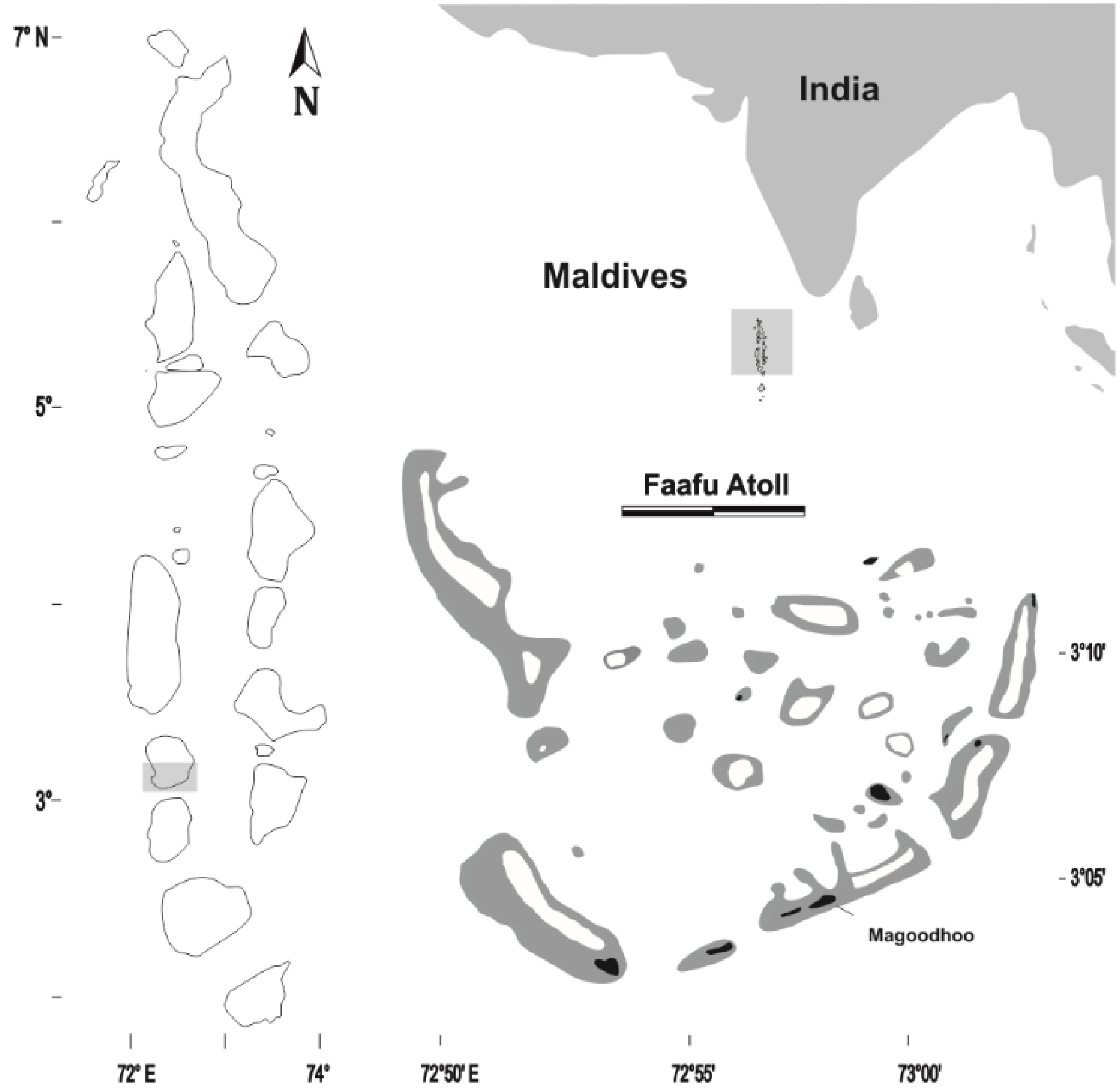

Figure S3. Localization of the experimental site. Schematic map of the localization of the Magoodhoo Island, Faafu Atoll, Republic of Maldives where the experiments were placed.
