# Supplementary material for: Treatment of Coral Wounds by Combining an Antiseptic Bilayer Film and an Injectable Antioxidant Biopolymer
Source: Sci Rep. 2020 Jan 22;10:988. doi: 10.1038/s41598-020-57980-1 (PMC6976594; doi:10.1038/s41598-020-57980-1)
Supplement: Supplementary file 2 — Supporting Information. [file 41598_2020_57980_MOESM2_ESM.docx]

|  |  |  |  |  |  |  |
| --- | --- | --- | --- | --- | --- | --- |
| **Injury condition** |  | **Coverage** |  | **General condition** |  | **Progression** |
|  |  |  |  |  |  |  |
| 0 |  | 0 |  | healthy |  | stable |
| 1 |  | 25 |  | not healthy (edges) |  | slighty increase |
| 2 |  | 50 |  | not healthy |  | huge increase |
| 3 |  | 75 |  | aggravation |  | tissue regrowth |
| 4 |  | 100 |  | treatment not efficent |  | total tissue regrowth |
|  |  |  |  |  |  |  |
| **Colony Health** |  | **Muco** |  | **Bleaching** |  | **Necrosis** |
|  |  |  |  |  |  |  |
| 0 |  | none |  | none |  | none |
| 1 |  | slighty |  | slighty pale (partial) |  | slighty close tips |
| 2 |  | diffused |  | pale (all colony) |  | diffused (other parts) |
| 3 |  | heavily diffused |  | white |  | total (all colony) |
| 4 |  | death |  | death |  | death |
|  |  |  |  |  |  |  |
| **Bio-paste condition** |  | **Hardening** |  | **Dissolution** |  | **Adhesion** |
|  |  |  |  |  |  |  |
| 0 |  | not applicable |  | not applicable |  | none |
| 1 |  | soft |  | istant dissolution |  | doubt application |
| 2 |  | semi rigid |  | rapid dissolution (24H) |  | not perfect |
| 3 |  | rigid |  | slow dissolution (10 gg) |  | adhesion lesion/no tissue |
| 4 |  | hard (plastic like) |  | no dissolution |  | adhesion lesion and tissue |
|  |  |  |  |  |  |  |
|  |  |  |  |  |  |  |
